# Study on the Flow Field Distribution in Microfluidic Cells for Surface Plasmon Resonance Array Detection

**DOI:** 10.3390/ma17102426

**Published:** 2024-05-17

**Authors:** Wanwan Chen, Jing Li, Peng Wang, Shuai Ma, Bin Li

**Affiliations:** 1 Department of Precision Instrument, Tsinghua University, Beijing 100084, China; peng@163.com (P.W.);; 2 College of Mechanical Engineering, Yangzhou University, Yangzhou 225012, China; 3 College of Environmental Science and Engineering, Yangzhou University, Yangzhou 225012, China; 4 Research Institute of Tsinghua, Pearl River Delta, Guangzhou 510530, China

**Keywords:** microfluidic cells, SPR array detection, finite element simulation, flow field

## Abstract

This research is dedicated to optimizing the design of microfluidic cells to minimize mass transfer effects and ensure a uniform flow field distribution, which is essential for accurate SPR array detection. Employing finite element simulations, this study methodically explored the internal flow dynamics within various microfluidic cell designs to assess the impact of different contact angles on flow uniformity. The cells, constructed from Polydimethylsiloxane (PDMS), were subjected to micro-particle image velocimetry to measure flow velocities in targeted sections. The results demonstrate that a contact angle of 135° achieves the most uniform flow distribution, significantly enhancing the capability for high-throughput array detection. While the experimental results generally corroborated the simulations, minor deviations were observed, likely due to fabrication inaccuracies. The microfluidic cells, evaluated using a custom-built SPR system, showed consistent repeatability.

## 1. Introduction

Surface plasmon resonance (SPR) sensing technology represents a cutting-edge optical technique characterized by its label-free, real-time, and high-sensitivity attributes, making it pivotal in a broad realm of applications ranging from molecular diagnostics to environmental monitoring [1,2,3]; therefore, it can be very useful for further development for further development of biosensing and bioelectronic methods [4,5]. Over decades, SPR has evolved into an indispensable tool for the investigation of biomolecular interactions, gaining widespread adoption across various sectors, including biochemical research, clinical diagnostics, and pharmaceutical development [6,7,8,9,10]. Central to the function of commonly deployed prism-based SPR biosensors is the SPR sensing unit, comprising a prism, a sensing chip, and an integral microfluidic system [11,12]. Within this setup, microfluidic cells play a critical role by housing the sample solution and facilitating adequate interaction time between analytes and target molecules on the sensing film, thus enhancing reaction efficacy [13,14]. To achieve precise detection, it is crucial to design the flow chamber to ensure a uniform solution flow rate across each target site, thereby minimizing the occurrence of errors attributable to differential residence times at various sensor locations [15,16]. The uniform flow maintained by the microfluidic chamber is essential for consistent and accurate detection outcomes [17,18].

The study of flow dynamics within these micro-sized cells has attracted significant scholarly interest [19,20,21]. Innovations such as the novel low-cost, multiparametric, stand-alone LSPR imaging instrument presented by Rampazzi et al. [22] and the rapid, label-free SPR instrument for multiplex nucleic acid detection proposed by Wang et al. underscore the ongoing advancements in the field [23,24,25]. Previous research has examined the implications of mass transport and interactions on analyte distribution extensively, revealing strategies to counteract the uneven distribution of analytes due to variable mass transport rates, such as modulating the flow rate or adjusting the density of receptor immobilization [26,27,28,29,30,31,32,33,34,35].

Recent advancements have further refined SPR technology through the integration of numerical analysis with experimental methodologies to optimize the design of microfluidic channels. These enhancements are informed by computational fluid dynamic (CFD) simulations that meticulously analyze flow structures, species concentration, and mixing efficiency within these systems [36,37,38]. This continuous development has notably improved the performance of SPR sensors, particularly in enhancing sensitivity and throughput.

This article delves into the critical issue of achieving uniformity in SPR array detection by marrying numerical analysis with empirical measurements. Leveraging fluid dynamics principles, it utilizes numerical simulations to explore the flow field distribution within microfluidic cells. The insights gained from these simulations have facilitated the design of microchannels optimized for SPR sensor array detection. Building upon these findings, a novel microfluidic system has been designed and fabricated. Moreover, by juxtaposing the results from numerical simulations with those from SPR detection experiments, this study delineates effective design strategies, thereby providing substantial guidance for the engineering of microfluidic cells.

## 2. Theory, Methodology, and Results of Simulation Studies

### 2.1. Basic Simulation Methods

#### 2.1.1. Simulation Model of Microfluidic Cells

A typical SPR sensing unit is depicted as follows. Probe molecules are anchored onto the gold film sensing surface through a coupling layer. When the solution containing the sample to be tested flows through the microfluidic cell, it concurrently passes over the sensing surface. In this process, the analyte molecules within the solution interact with the probe molecules that are affixed to the gold film. This interaction is generally reversible, meaning that while analyte molecules bind to probe molecules to form complexes, some of these complexes may dissociate back into separate analyte and probe molecules. The formation of complexes between analyte and probe molecules leads to an increase in mass on the sensing surface, which, in turn, causes a change in the refractive index. SPR is capable of sensing these changes in real time, thereby deriving information about biomolecular interactions, such as binding and dissociation rates, $k_{a}$, $k_{d}$.

The features of array detection include the following: The sensing chip is organized in an array format, with each array spot capable of attaching different “probes”. As the sample solution flows across the arrayed sensing surface, the analyte molecules within the solution can couple or react with the probe molecules located at specific array spots. By affixing a unique probe to each sensing spot, it becomes possible to concurrently analyze multiple types of analyte molecules as they react with the probes at various sensing spots in a single assay, thus facilitating high-throughput detection.

**Figure 1:**
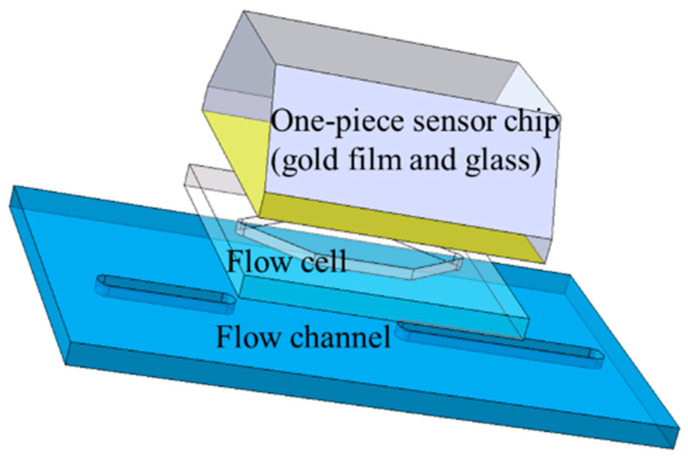
Typical SPR sensing system.

**Figure 2:**
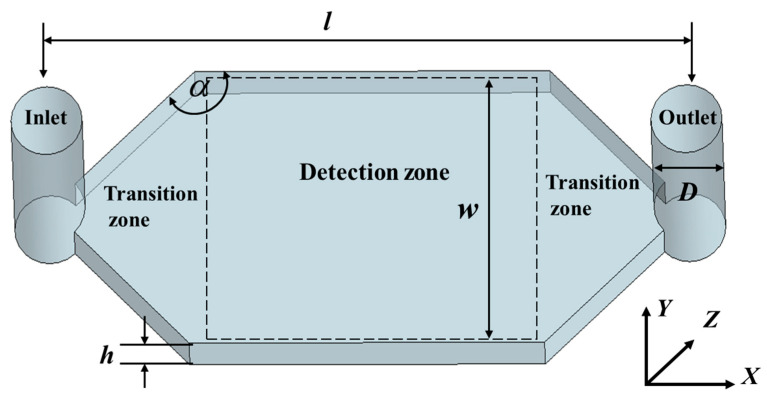
Schematic diagram of fluid cell unit model.

The most commonly used detection chip, as illustrated in Figure 1, comprises three main components: the inlet and outlet, the transition section, and the detection area. For SPR detection, it is crucial for the array sensing points to be situated within the detection area, emphasizing the importance of analyzing the flow field’s uniformity in this zone. The dimensions of the gold film on the sensitive chip utilized by the laboratory’s homemade SPR sensor array are 22 mm by 28 mm. The target array (detection area) spans an area of less than 11 mm by 11 mm and is centrally positioned within the flow cell. The design of the flow cell must ensure that it encompasses the detection area without exceeding the boundaries of the gold film. This paper presents various flow cell models designed for analytical testing, illustrating the placement of the array detection microfluidic cell within the SPR sensing unit. Positioned above the gold film, it primarily includes the solution inlet and outlet, the transition section, and the detection area. The design parameters specified in this study include cylindrical channels for the inlet and outlet with a diameter of D=1mm, a center-to-center distance of l=9mm, a flow cell width and height of w=4mm and h=0.4mm. Assuming the angle of the transition section is α, the detection area’s size, as depicted in Figure 2 for typical flow cells, significantly influences the uniformity of the flow field within the detection zone, which is critical for array detection. The detection area *s* is calculated as

(1) $$s=w\times \left[l-w\times \tan(\alpha -90{}^{\circ})\right]\;\;(90^{{}^{\circ}}<\alpha <150^{{}^{\circ}})$$

It is evident that the angle α not only determines the area of the detection zone but also affects the uniformity of the flow velocity distribution, which is directly related to the array’s size. Furthermore, it dictates the volume of the microfluidic cell, significantly impacting the consumption of samples and reagents.

Additionally, the flow rate has a significant impact on the flow velocity distribution within the microfluidic cell. Only after a thorough investigation of the effects of these two parameters on the flow velocity distribution can a scientific and reasonable design be achieved. Moreover, to ensure that the microfluidic cell is reliably sealed, a minimum of 5 mm should be reserved along the edges of the cell. Flow pattern analysis indicates that the flow velocity near the walls of the microfluidic cell is much smaller than that in the center area. To ensure good uniformity of flow velocity at each array point, the sensing points should be positioned a suitable distance away from the walls of the microfluidic cell while also considering a larger range of choices for the angle α, with 90° ≤ α ≤ 137° being preferable. This range allows for enhanced flow dynamics and uniformity, which are essential for the accurate performance of SPR-based detection systems.

#### 2.1.2. Boundary Conditions and Numerical Model Conditions

The characteristics of the fluid in the microfluidic cell determine the consistency of the flow velocity distribution and, thus, need to be considered comprehensively. The fluid Reynolds number in the microfluidic cell is

(2) $$R_{e}=Lv\rho /\mu$$

In the formula, L represents the maximum characteristic length, v is the average velocity of the fluid, ρ is the density of the fluid, and μ is the viscosity of the fluid. Simplifying the analysis by assuming the fluid in the microfluidic cell is pure water at room temperature, the density is ρ = 1000 kg/m^3^, μ = 10^−3^ Pa⋅s. The flow rates from syringe pumps or peristaltic pumps are generally in the range of several microliters to a few milliliters per minute. With a flow rate of 500 μ L/min, L=D=1mm, the $R_{e}\approx 2.3$ << 2100. Therefore, the microfluid in the microfluidic cell is laminar, satisfying the continuity Equation (3) and the momentum Equation (4) of the Navier–Stokes equation:

(3) ∇V=0

(4) $$\rho \left(\frac{\partial V}{\partial t}+V\nabla V\right)=-\nabla P+\rho g+\mu \nabla^{2}V$$

In the microfluidic cell, the velocity field is a steady flow field, and the influence of gravity can be neglected. Thus, the momentum Equation (4) can be simplified to

(5) $$\nabla P=\mu \nabla^{2}V$$

where μ represents the viscosity coefficient, P is the pressure, and V is the velocity vector.

Furthermore, boundary conditions such as the inflow, outflow, and wall slip of the flow field also need to be considered. Among these, the inflow rate at the microfluidic cell inlet is typically the maximum flow rate used, which is $Q_{\max}=500\;\mu L/\min$; at the microfluidic cell outlet, the flow field can be considered fully developed, meaning $P_{out}=0$. To assess the wall conditions, the Knudsen number for the fluid is calculated as follows:

(6) $$K_{n}=\lambda /L$$

where λ represents the mean free path of the fluid molecules. The mean free path of water molecules is approximately $2.14\times 10^{-7}$ m. Therefore, the Knudsen number $K_{n}=2.14\times 10^{-4}<10^{-3}$, which satisfies the no-slip boundary condition.

In this study, COMSOL Multiphysics software 6.0 was employed to simulate flow cells with diverse geometries guided by well-established control equations and boundary conditions. Initially, geometric models of these fluid cells were developed using SOLIDWORKS and subsequently imported into COMSOL Multiphysics for comprehensive simulation analysis. A free tetrahedral mesh approach was used for the simulation mesh, with element sizes in the larger chamber configurations tailored between 0.18 mm and 0.008 mm. It was noted that additional refinement of the mesh did not significantly alter the simulation outcomes, confirming this mesh density was optimal for capturing the essential dynamics of the flow.

The simulations utilized the ‘Laminar Flow’ and ‘Transport of Diluted Species’ modules in COMSOL, applying a no-slip condition at all boundaries to closely replicate physical boundary interactions. The inlet conditions were determined by the preset fluidic flow rate, while the outlet was maintained at a pressure condition of 1 atmosphere, adjusted for hydrostatic pressure and backflow prevention. Additionally, dynamic viscosity was set according to room temperature (293.15 K) to accurately reflect standard laboratory conditions.

### 2.2. Discussion of Simulation Results

#### 2.2.1. Impact of Contact Angle on Flow Field Uniformity

Simulations were conducted on the fluidic cell model illustrated in Figure 2, focusing on the analysis of the flow field distribution at the cross-section at h/2. Moreover, incorporating Equation (1) and taking into consideration various factors, contact angles of 115°, 125°, and 135° were chosen for further simulations. The inlet flow rate was set to 100 μL/min to analyze the impact of different contact angles on the uniformity of the flow field distribution within the detection area.

**Figure 3:**
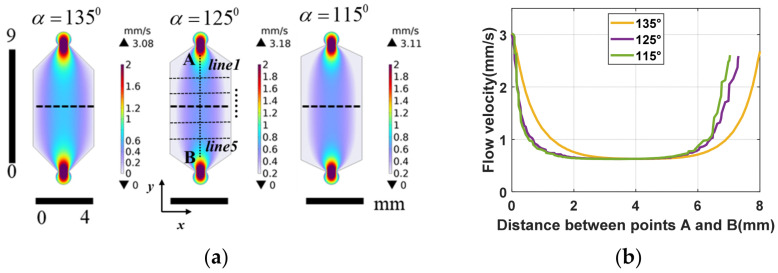
Simulation results of surface flow velocity distribution at different contact angles: (**a**) simulation results of surface flow velocity distribution of microfluidic cell at 100 μL/min; (**b**) simulation results at line AB in (**a**).

When the solution is in the process of entering the fluid cell, the corresponding flow rate decreases due to the sudden expansion of the flow path, and the opposite is true for the outlet, so it is not taken into account during the calculation of the flow field distribution. In Figure 3, it is shown that the flow velocity is higher in the region close to the inlet and outlet and smaller near the wall, and there is a more consistent distribution of flow velocity in other regions.

**Figure 4:**
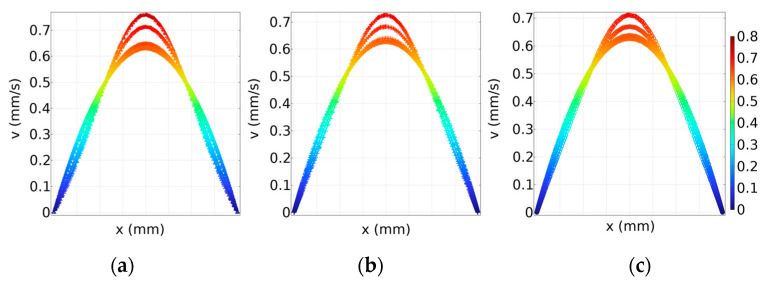
Simulation results of cross-sectional surface flow velocity distribution at different contact angles: (**a**) α = 135°; (**b**) α = 125°; (**c**) α = 115°.

To further study the flow field distribution from the inlet to the outlet in the detection area, five cross-sections along the length (line AB) of the detection area were selected, sequentially labeled line 1 through line 5 in the y-coordinate, as shown in Figure 3a. Subsequently, a quantitative analysis was performed on how different contact angles affect the area of uniform flow velocity within the detection area, with the flow velocity distribution curves for cross-sections shown in Figure 4, and the *x*-axis represents the position along the width of the fluidic cell.

Furthermore, due to the presence of stationary walls and the fluid’s viscosity, the velocity decreases closer to the walls, resulting in varying flow velocities across different locations within the microfluidic cell. By taking the surface velocity in the range of 0.45~0.75 mm/s, the velocity distribution area is about 11.15 mm^2^, 10.95 mm^2^, and 12.09 mm^2^, corresponding to the contact angles of 115°, 125°, and 135°, respectively.

The difference in flow velocity distribution is defined as follows: $(v_{\max}-v_{\min})/v_{\max}$. The percentage differences in the flow velocity at contact angles of 135°, 125°, and 115° are 15.71%, 13.64%, and 11.83%, respectively. These results indicate that larger contact angles tend to result in a poorer uniformity of the flow velocity distribution. Subsequently, the surface area was calculated, and the consumption flow rate of the fluid cell was determined by multiplying the area and depth. According to the Table 1, smaller microfluidic cell volumes consume less solution, and the area calculation does not include the inlet and outlet areas. The occurrence of this scenario can be attributed to the detection area being closer to the inlet and outlet at 115°, resulting in larger changes in flow velocity and a longer time needed for the flow velocity to stabilize. Clearly, to achieve a larger area of uniform flow field distribution, the contact angle should be 135°.

#### 2.2.2. Effects of Flow Velocity on Flow Field Uniformity

**Figure 5:**
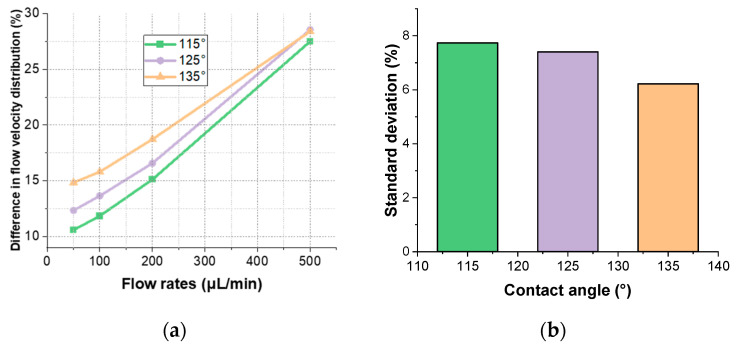
Percentages of different flow rates with different contact angles: (**a**) distribution of flow rates with different contact angles for different flow rates; (**b**) standard deviation of flow velocity distributions with different contact angles for different flow rates.

During experiments, different flow rates are selected as required. To choose the optimal design parameters, it is necessary to analyze the distribution of flow velocity within the microfluidic pool under various flow rates. Typically, flow rates are set at 50 μL/min, 100 μL/min, 200 μL/min, and 500 μL/min. Simulations are conducted with these four flow rates, and the results of the flow velocity distribution are presented in Figure 5.

In Figure 5a, the flow rate percentage variations within the microfluidic cells for three examined contact angles at flow rates of 50 μL/min, 200 μL/min, and 500 μL/min are 10.57%, 15.11%, 27.50% for a contact angle of 115°; 12.32%, 16.57%, 28.55% for a contact angle of 125°; and 14.82%, 18.71%, 28.40% for a contact angle of 135°, respectively. Figure 5b shows that the standard deviations of the flow velocity distribution are 7.73%, 7.40%, and 6.21% for these contact angles across varying flow rates. As the flow rate increases, the percentage distribution of flow velocity at each contact angle also rises, with larger contact angles displaying higher percentage distributions, indicating that a larger contact angle facilitates a higher volume of fluid traversing the cell per unit time. The increase in the flow velocity distribution percentage becomes more significant, especially when the flow rate rises from 200 to 500 μL/min. At the highest flow rate of 500 μL/min, the flow velocity distribution percentages for different contact angles converge, reaching a nearly equivalent value of about 29%. These findings demonstrate that the contact angle significantly impacts the flow velocity distribution, and an increase in flow rate notably enhances the flow velocity distribution percentage across all contact angles, particularly at higher flow rates. Consequently, for practical applications, a flow rate of less than 200 μL/min should be considered.

#### 2.2.3. Effects of Micro-Pillar Structured Microfluidic Cell Detection Arrays

Simply altering the length of the transition section has a relatively limited impact on the uniformity of lateral velocity. The length of the flow cell is often constrained by various factors, such as the detection area, and cannot be increased excessively. Therefore, to enhance the uniformity of flow velocity through the disturbance of the fluid motion, the introduction of turbulence structures within the flow cell is considered. In the subsequent research, this paper takes a contact angle of 135° as an example and introduces a micro-pillar array turbulence structure in its transition area. The effects of micro-pillar dimensions, quantity, and distribution on the flow field were investigated.

(1) Impact of micro-pillar dimensions on the flow field distribution in the detection area

**Figure 6:**
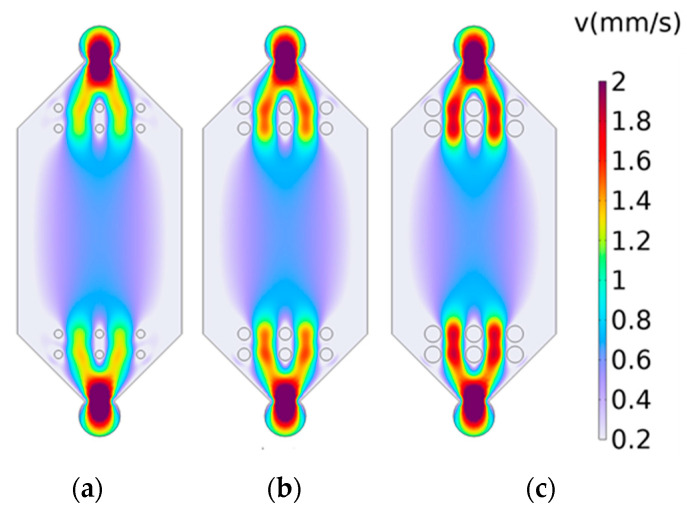
Simulation results on the effect of micro-pillar sizes on the flow field distribution: (**a**) radius of 0.1 mm; (**b**) radius of 0.15 mm; (**c**) radius of 0.2 mm.

Circular micro-pillar arrays were added to the transition section of the flow cell, symmetrically arranged on both sides. Based on prior experimental experience, a 2 × 3 circular micro-array was added to the transition section of the model. Taking the center point of the flow cell as the origin, the impact of different micro-pillar sizes was examined by conducting simulations with micro-pillar of 0.1 mm, 0.15 mm, and 0.2 mm and without the addition of micro-pillar structures. The simulation results (shown in Figure 6) indicated that the introduction of micro-pillar arrays has a limited effect on improving the axial velocity distribution, which was already near ideal and did not significantly affect objectives. However, the introduction of micro-pillar arrays had a significant effect, with arrays having radii of 0.15 mm and 0.2 mm showing considerable improvements in the uniformity of velocity distribution. Among these, the flow cell with added micro-pillars of 0.15 mm radius exhibited a closer-to-ideal distribution of both axial and lateral velocities.

(2) Impact of micro-pillar distribution on the flow field distribution in the detection area

**Figure 7:**
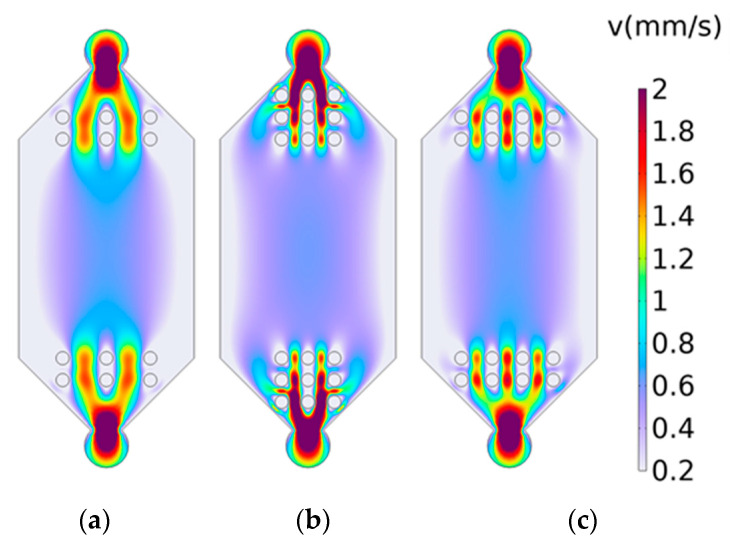
Simulation results of the effect of micro-pillar distribution on the flow field distribution: (**a**) 2 × 3; (**b**) 3 × 3; (**c**) 2 × 4.

Previous studies have demonstrated that micro-pillar arrays with a radius of 0.15 mm exhibit a positive effect on the uniformity of the flow field. Therefore, in the following experiment, micro-pillars of this size were utilized. To compare the impact of the number of micro-pillars on the flow field distribution, simulations were conducted with 2 × 3 (two columns and three rows), 2 × 4, and 3 × 3 micro-array configurations, all with 1 mm spacing in the y axis. Compared to cases without micro-pillar arrays, the results indicated that the axial velocity distribution was more uniform in the 2 × 3 and 2 × 4 configurations, as shown in Figure 7a,b. In contrast, the lateral velocity distribution was more uniform in the 3 × 3 configuration, as depicted in Figure 7c. These findings suggest that increasing the number of columns in the array enhances the uniformity of the lateral flow velocity but may compromise the axial flow velocity’s stability. Conversely, adding more rows appears to have a minor effect, with the three-row configuration exhibiting slightly improved performance.

In the conducted investigation, utilizing a micro-pillar array configuration with a 0.15 mm radius arranged in a 2 × 3 format, the impact of varying micro-pillar spatial distributions on the fluid dynamics was explored. The study considered three distinct spacing scenarios with inter-row and inter-column distances set at 0.6 mm, 0.8 mm, and 1 mm, respectively. The findings from the simulations suggest that micro-pillar arrays facilitate lateral flow field stabilization. An increment in the separation between rows within the micro-array modestly ameliorates the axial flow dynamics but adversely affects lateral flow coherence. Altering the spacing between columns yielded a negligible influence on overall flow behavior. From this investigation, several key insights emerge:

(1) Across different boundary configurations, changes in the flow dynamics within the detection zone are minimal; however, the flow velocity’s stability within the detection zone exhibits significant variability contingent on the differential length from the inlet to the outlet. This variability is closely related to the differential length from the inlet to the outlet and is influenced by the contact angle.
(2) The integration of an optimally sized transition section within the microfluidic environment is conducive to enhancing the homogeneity of the fluid flow field.
(3) Incorporating micro-structural arrays to induce flow turbulence within flow cells can improve flow uniformity, though it may lead to vortex formation near the inlet.

Thus, the strategic incorporation of micro-structural arrays within the transition section of the flow cell proves advantageous for facilitating high-throughput detection methodologies. These outcomes offer critical insights into the architectural design of flow cells in the context of SPR sensor applications. Considering factors such as flow rate distribution error, sample consumption, and the standardized variance of flow rate distribution at different velocities—and taking into account the machining difficulty—the flow cell with a contact angle of 135 degrees has been chosen for subsequent experimental validation.

## 3. Experimental Study of Flow Distribution

### 3.1. Mic-PIV Experiment

The tracer particles used are small in volume, with a diameter of only 1 μm, necessitating the use of high magnification objectives to accurately observe the motion of the particles. This results in the field of view for measurements taken using micro-PIV being limited to only 0.9 × 0.5 mm^2^. Given that the detection area of the microfluidic cell is 10 × 10 mm^2^, to capture images of the entire area, it is necessary to collect images field by field and then stitch these images together to present the overall flow velocity distribution within the fluid. The micro-PIV system used in this experiment utilizes tracer particles that are 1 μm diameter polystyrene fluorescent beads (Sourced from Duke, USA, purchased from Thermo Fisher Scientific Inc., Waltham, MA, USA) with a specific gravity of 1.055 and a volume concentration of 0.07%. These tracer particles emit red fluorescence with a wavelength of 610 nm when excited by green light, which is then recorded by a CCD camera after passing through the objective and a filter. The flow velocity at the position to be measured in the fluid pool can be obtained by extracting data along a line parallel to the *X*-axis.

**Figure 8:**
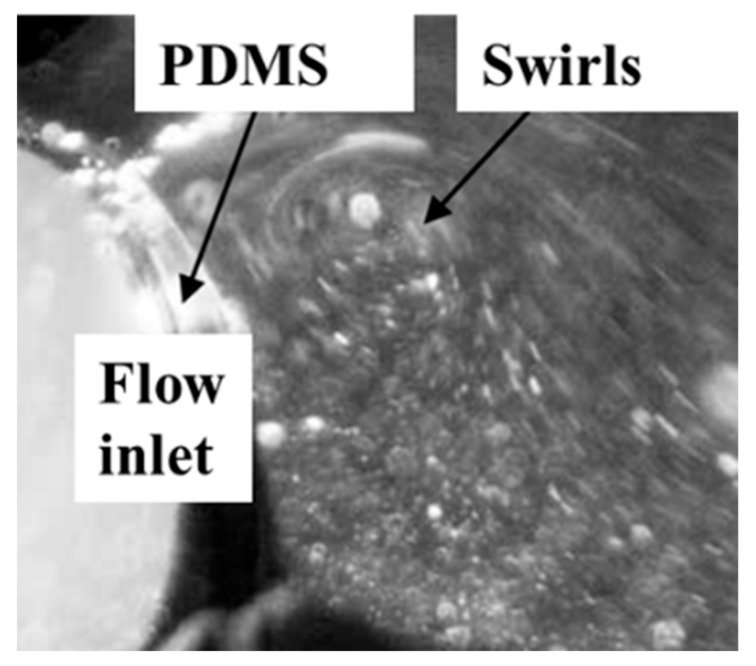
Micrographs of fluid flow conditions at the inlet of a microfluidic cell.

Through microscopic observation, the fluid flow at the entrance of the microfluidic cell is examined, as depicted in Figure 8. It is observed that the entrance edge is uneven, leading to non-uniform fluid entry into the microfluidic cell and resulting in vortex formation within. Consequently, two potential outcomes are identified: firstly, the flow velocity distribution on the same longitudinal cross-section within the microfluidic cell might be irregular, precluding uniform distribution and impacting the consistency of array detection; secondly, the microfluidic cell, initially filled with air, experiences asymmetric flow patterns due to vortices as the fluid enters. This asymmetry could prevent one side of the microfluidic cell from being completely filled with fluid while allowing fluid to reach the outlet on the opposite side, thus failing to expel all air and leaving residual air bubbles that can affect detection.

The underlying cause of this phenomenon is traced back to the fabrication process of the microfluidic system. During the formation of the microfluidic cell’s inlet and outlet, a slight gap exists between the insertion needle and the mold hole. When the liquid PDMS mixture is injected into the mold and heated, a small amount of PDMS leaks out through these gaps. After the PDMS has fully solidified, removal of the insertion needle and demolding occurs without adequately cleaning the leaked PDMS. Properly removing the excess PDMS smoothens the entrance edge of the microfluidic cell, ensuring uniform fluid entry. This underscores the critical importance of process control in the manufacturing of microfluidic systems.

**Figure 9:**
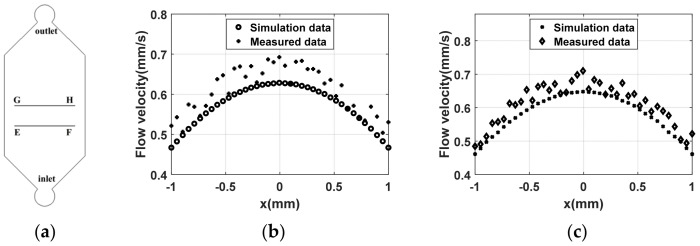
Comparison of simulation and experimental results: (**a**) schematic diagram of cross-section location; (**b**) flow velocity distribution in line EF; (**c**) flow velocity distribution in line GH.

Next, the simulated data are compared with the data measured using PIV. The flow rate is set to 100 μL/min, and comparisons are made at the central line position within the microfluidic cell shown in Figure 9a.

The figures reveal slight discrepancies between the experimental measurements and the simulation results at various positions within the microfluidic cell, yet the trends of both measurements and simulations are fundamentally aligned. From Figure 9b, it is noted that, at x = 0, the simulated flow velocity is 0.63 mm/s, compared to the measured flow velocity of 0.68 mm/s, indicating close agreement; at x = −0.5 mm, the simulated flow velocity is 0.58 mm/s, with a measured flow velocity of 0.63 mm/s, showing a slightly larger difference; at x = 0.5 mm, the simulated flow velocity is 0.57 mm/s, and the measured flow velocity is 0.61 mm/s, both of which are also closely matched. Figure 9c shows that at x = 0, the simulated flow velocity is 0.64 mm/s versus a measured flow velocity of 0.68 mm/s, which are relatively close; at x = −0.5 mm, the simulated flow velocity is 0.59 mm/s, with a measured flow velocity of 0.65 mm/s, indicating a slightly larger difference; at x = 0.5 mm, the simulated flow velocity is 0.61 mm/s, and the measured flow velocity is 0.63 mm/s, both of which are quite close. Therefore, it can be concluded that the experimental data are slightly higher than the simulation data, particularly for x > 0. Measurement of the fabricated microfluidic cell using a white light interferometer reveals that, due to manufacturing errors, the depth of the microfluidic cell on the x > 0 side is small, with the minimum depth being 405 μm, while the depth on the x < 0 side is greater, reaching up to 408 μm. In fact, a smaller depth corresponds to a smaller cross-sectional area; conversely, a greater depth results in an increased cross-sectional area. At a constant flow rate, a smaller cross-sectional area leads to a faster flow velocity. Clearly, manufacturing errors are the primary cause of the minor discrepancies between the experimental and simulated results. In summary, the measurement and simulation results are fundamentally consistent, validating the feasibility of the design methodology. The simulation results can, therefore, guide the design process.

### 3.2. SPR Array Detection Experiments

**Figure 10:**
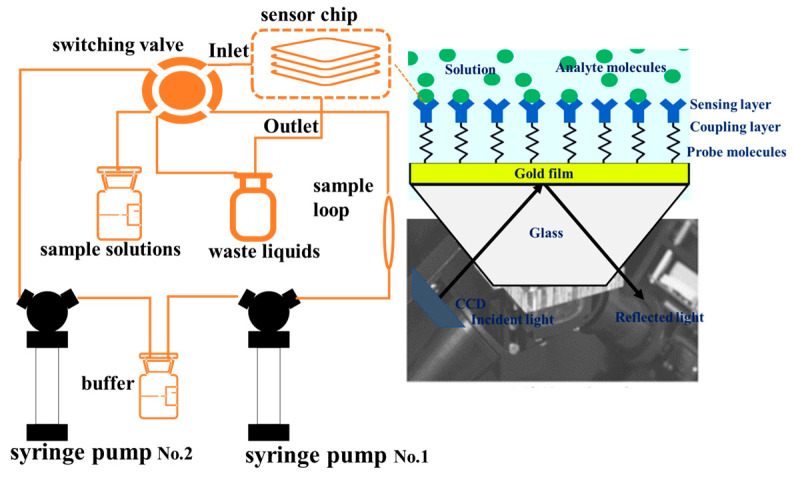
Experimental setup for SPR array detection consists of the microfluidic system.

The designed microfluidic cell is integrated with the existing laboratory-developed array-based SPR sensing system to form a complete SPR detection instrument with a microfluidic system. This integration allows for precise temporal control of sample and reagent flow over the sensing surface, determining the consistency of the array biomolecular reactions and affecting detection reliability. The experimental microfluidic system includes microvalves, microchannels, and microfluidic cells. The sensing surface of the SPR chip faces the microfluidic cell, with these components being tightly sealed together. To meet these requirements, special processes are employed, utilizing PDMS to fabricate the microvalves, microchannels, and microfluidic cells. The control of air valves is implemented on Plexiglass (PMMA), and both components are integrated together. The completed assembly of the experiment is shown in Figure 10.

The PDMS base of the microfluidic cell can be disassembled and reassembled, allowing for the installation of microfluidic cells with different shapes and structures into the SPR array detection device for experimentation.

#### 3.2.1. Preliminary Experiment for Detection

SPR sensing is based on the changes in the refractive index on the surface of a gold film. When solutions with different refractive indices flow over the gold film, corresponding signals are generated. The refractive index of a NaCl solution, for example, is directly proportional to its concentration. An experiment using a 5% salt water solution involving a single injection was conducted to observe the phase change curve during a single injection. It is observed that the signal experiences two jumps during the injection process. By examining the images captured before, during, and after these jumps, it is found that the cause of the jumps is the entry and exit of bubbles in the reaction fluidic cell. An analysis of the reasons for bubble entry into the fluidic cell points to two factors:

(1) Inconsistency in the inner diameter of the tubing, leading to the fragmentation of bubbles into several smaller segments;
(2) A significant discrepancy between the set volume of the tubing and the actual volume, resulting in bubbles not being completely expelled through the bypass. The bubble issue can be resolved by replacing the tubing and adjusting its volume and tolerance appropriately.

After stabilizing the system setup, 5% and 0.9% concentrations of NaCl solution were selected as sample solutions, with deionized water serving as the buffer. The flow rate was set to 100 μL/min. The experimental procedure is as follows:

(1) Inject deionized water into the microfluidic cell; continue for 150 s to establish a baseline.
(2) Inject 5% NaCl solution; continue for 180 s to measure its change in refractive index.
(3) Switch back to deionized water; continue for 300 s.
(4) Inject 0.9% NaCl solution; continue for 180 s.
(5) Reinject deionized water.

**Figure 11:**
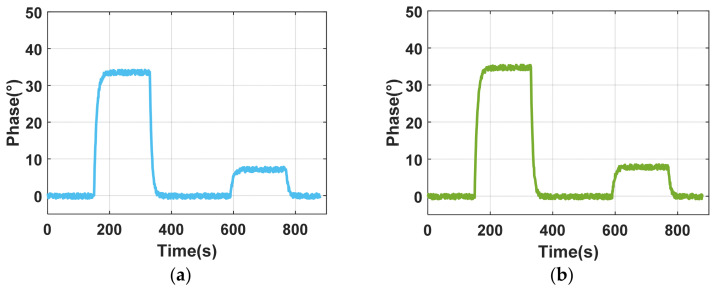
Corresponding changes in different detection areas: (**a**) detection area A; (**b**) detection area B.

The above process is completed automatically via computer-controlled microvalve switching and sampling mechanisms. Two different positions on the sensing surface are selected, and the phase changes within 10 × 10 pixels at various positions within the corresponding images captured by CCD are averaged. The experiment was conducted more than three times, and the results of any one iteration are presented, as illustrated in Figure 11. The results indicate a good consistency between the two areas, demonstrating that the design of the microfluidic cell meets the requirements of SPR array detection.

#### 3.2.2. Repeatability Experiments

**Figure 12:**
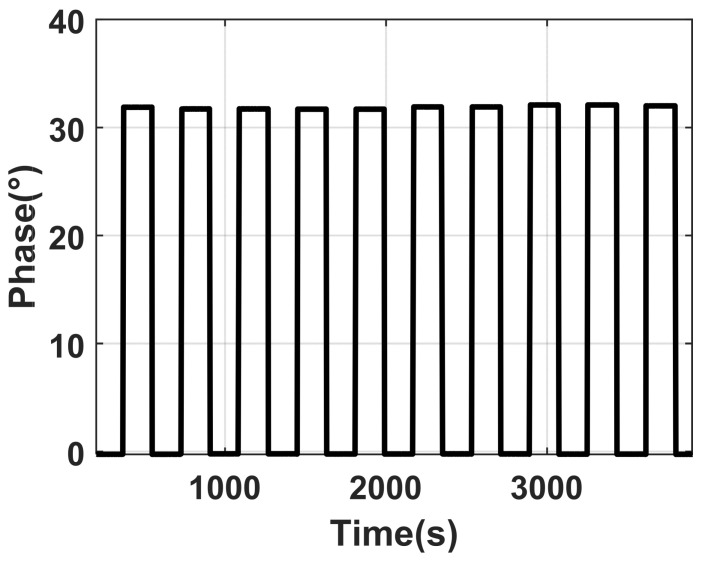
Experimental results of repeated switching.

To conduct a biological experiment, it is often necessary to frequently alternate microvalves within a span of several minutes to facilitate the substitution of solutions within the microfluidic cell; thus, repeatability becomes a crucial factor in safeguarding the precision and comparability of the experimental outcomes. On the apparatus for dual-differential interference imaging SPR detection, the repeatability of the system is evaluated by conducting successive measurements of changes in the refractive index of solutions. The protocol of these experiments involved alternating injections of deionized water and 5% NaCl solution into the microfluidic cell at a constant flow rate of 100 μL/min for a total of 10 cycles. A specific region of interest, i.e., a 10 × 10 pixels area within the central detection zone on the sensing surface, was chosen for computational analysis. The results are illustrated in the accompanying figure (Figure 12). The analysis yielded a standard deviation of 1.5 s for the amplitude’s ascent and descent timings. The metric for repeatability is expressed as the relative standard deviation of the response values across 10 iterations, amounting to approximately 1.6%.

## 4. Conclusions

This research facilitated the development of an advanced microfluidic cell integrated with a custom-built sample introduction system, significantly enhancing the SPR array sensing apparatus. This integration optimized the fluid flow velocities, achieving a more uniform flow velocity distribution within the microfluidic cell at an optimal contact angle of 135°. The alignment with simulation predictions not only confirms the simulations’ efficacy in guiding design enhancements but also underscores the importance of precise fabrication processes in achieving high-quality flow velocity distributions.

Furthermore, experimental validations demonstrated that the redesigned microfluidic system supports the SPR array in detecting saline and biomolecular interactions with remarkable precision. The system achieved a standard deviation of just 1.6% in the sample introduction, highlighting its robustness and potential to significantly advance biosensing applications. These improvements in the microfluidic setup represent advancements in the practical deployment of SPR technology for biosensing to a certain degree.

## Figures and Tables

**Table 1:** Variations in microfluidic cells surface and flow velocity distribution under different contact angles.

Contact Angle α(°)	135	125	115
Microfluidic cell area (mm^2^)	30.50	29.23	27.61
Flow velocity distribution (mm/s)	0.757~0.638	0.726~0.627	0.710~0.626

## Data Availability

Data are contained within the article.
